# Parkin deficiency prevents chronic ethanol-induced hepatic lipid accumulation through β-catenin accumulation

**DOI:** 10.1186/s12964-019-0424-5

**Published:** 2019-08-22

**Authors:** Dong Hun Lee, Mi Hee Park, Chul Ju Hwang, Youngsoo Kim, Dae Yeon Hwang, Sang Bae Han, Jin Tae Hong

**Affiliations:** 1 College of Pharmacy and Medical Research Center, Chungbuk National University, 194-31 Osongsaengmyeong 1-ro, Osong-eup, Heungduk-gu, Cheongju, Chungbuk 28160 Republic of Korea; 2 Department of Biomaterial Science, Pusan National University, 2, Busandaehak-ro 63beon-gil, Geumjeong-gu, Busan, 46241 Republic of Korea

**Keywords:** Parkin, Ubiquitination, β-Catenin, Alcoholic fatty liver

## Abstract

**Background:**

Alcohol abuse and alcoholism lead to alcohol liver disease such as alcoholic fatty liver. Parkin is a component of the multiprotein E3 ubiquitin ligase complex and is associated with hepatic lipid accumulation. However, the role of parkin in ethanol-induced liver disease has not been reported. Here, we tested the effect of parkin on ethanol-induced fatty liver in parkin knockout (KO) mice with chronic ethanol feeding.

**Methods:**

Male wild type (WT) and parkin KO mice (10–12 weeks old, *n* = 10) were fed on a Lieber-DeCarli diet containing 6.6% ethanol for 10 days. Liver histological, biochemical, and gene-expression studies were performed.

**Results:**

Parkin KO mice exhibited lower hepatosteatosis after ethanol consumption. Because several studies reported that β-catenin is a critical factor in ethanol metabolism and protects against alcohol-induced hepatosteatosis, we investigated whether parkin changes β-catenin accumulation in the liver of ethanol-fed mice. Our results show that β-catenin was greatly accumulated in the livers of ethanol-fed parkin KO mice compared to ethanol-fed WT mice, and that parkin binds to β-catenin and promotes its degradation it by ubiquitination. Moreover, the β-catenin inhibitor IWR-1 abrogated the attenuation of ethanol-induced hepatic lipid accumulation by parkin deficiency in the livers of parkin KO mice and parkin siRNA-transfected human hepatic cell line.

**Conclusions:**

Parkin deficiency prevents ethanol-induced hepatic lipid accumulation through promotion of β-catenin signaling by failure of β-catenin degradation.

**Electronic supplementary material:**

The online version of this article (10.1186/s12964-019-0424-5) contains supplementary material, which is available to authorized users.

## Background

Excessive and chronic alcohol consumption leads to alcoholic liver disease such as alcoholic fatty liver, alcoholic hepatitis, cirrhosis, and hepatocellular carcinoma. Ethanol induces steatosis, which is characterized by the accumulation of fat in hepatocytes [1]. Several studies suggest that disruption of mitochondrial β-oxidation of fatty acids and dysregulation of lipid metabolism are significant factors in the chronic alcohol consumption-induced hepatic lipid accumulation [2, 3].

The β-catenin signaling pathway is associated with cell survival under oxidative stress as well as hepatic metabolic zonation [4, 5]. Lehwald et al. reported that β-catenin mediates hepatocyte protection in hypoxia or ischemia/reperfusion-induced oxidative injury of the liver [6]. Tao et al. also reported that Wnt/β-catenin signaling protects against oxidative stress-induced apoptosis in mouse liver [7]. These results suggest that β-catenin signaling may play a protective role in oxidative stress-induced liver injury. The β-catenin signaling is closely associated with obesity and lipogenesis [8]. In human bone marrow mesenchymal progenitor cells, β-catenin signaling agonist enhances adipogenensis as shown lipid accumulation but IWR-1, β-catenin signaling inhibitor, abrogate adipogenesis which is induced by adipogenic induction medium [9]. The β-catenin signaling pathway is also important for metabolic homeostasis in the liver [10]. Liver-specific β-catenin deficiency has been shown to increase susceptibility to diet-induced and ethanol-induced hepatosteatosis in mice [11, 12]. Furthermore, Wnt/β-catenin signaling is associated with mitochondrial biogenesis and insulin sensitivity [13]. These data suggest that β-catenin signaling play an important role as a regulator in hepatic mitochondrial function and lipid accumulation.

Parkin is a protein that is encoded by the *PARK2* gene, and it is a component of the multiprotein E3 ubiquitin ligase complex [14]. Parkin modulates several proteins that act in various physiological functions by degrading or modifying them [15]. *PARK2* mutation leads to autosomal recessive juvenile Parkinson’s disease (PD) [16]. Recently, several studies reported that dietary fat or obesity is associated with risk of PD; higher fat intakes and excess weight may be potential risk factors for PD [17, 18]. Thus, parkin may be associated with lipid metabolism. In some recent reports, parkin protects against liver injury or hepatic steatosis by acute ethanol consumption [19, 20]. However, other studies reported opposing data that parkin-deficient mice showed reduced body weight [21] and attenuation of a high fat diet-induced fat accumulation in adipose tissue and the liver [22]. Thus, relationship between parkin and lipid accumulation and its mechanism of action in the liver is unclear. Moreover, we observed that parkin-deficient mice showed attenuated hepatic steatosis by chronic ethanol consumption. Parkin is a component of the E3 ubiquitin ligase complex and β-catenin, which plays an important role in hepatic metabolism, is degraded by parkin [23]. Therefore, we investigated whether parkin deficiency may prevent chronic ethanol-induced hepatic lipid accumulation through changes in the β-catenin pathway in the liver.

## Methods

### Animals

The experimental treatments were carried out according to the guidelines for animal experiments of the Faculty of Disease Animal Model Research Center, Korea Research Institute of Bioscience and Biotechnology (Daejeon, Korea), as well as the Guidelines for the welfare and use of animals in cancer research [24]. The parkin knockout (parkin KO) mice (C57BL/6 background) were purchased from the Jackson Laboratory. For chronic ethanol consumption experiment, we used short-term NIAAA model [25, 26]. Briefly, male, age matched (12 weeks) parkin KO mice and wild type (WT, C57BL/6) mice were randomly divided into four groups (*n* = 10 per group), Each group (parkin KO and WT) of mice received 2 different types of liquid diets for 10 days: (1) WT mice fed standard diet with water (WT pair-fed); (2) parkin KO mice fed standard diet with water (KO pair-fed); (3) WT mice fed alcohol diet with ethanol (WT EtOH-fed); (4) parkin KO mice fed alcohol diet with ethanol (KO EtOH-fed). Ethanol comprised 35.8% of total calories in mice receiving ethanol. Liquid diets (pair-fed and EtOH-fed) were based upon the Lieber-DeCarli ethanol formulation and were purchased from DYETS Inc. (Bethlehem, PA). The ethanol concentration was kept thereafter at 6.6% for 10 days. On the morning of 11th day, acute ethanol (5 g/kg of body weight) administration into EtOH-fed mice for further elevation of blood alcohol levels and acute maltose dextrin (9 g/kg of body weight) administration into pair-fed mice for control were performed then sacrificed 9 h post gavage.

### Clinical analyses

Mice were anesthetized with an overdose of pentobarbital (100 mg/kg) and blood was taken by heart puncture. Serum levels of aspartate transaminase (AST), alanine transaminase (ALT) and the levels of triglycerides and cholesterols in the liver of mice were measured by using an automated analyzer (7080, Hitachi Ltd., Japan) at laboratory animal research center in Chungbuk National University.

### Mouse primary hepatocyte isolation and cell culture

The primary mouse hepatic cells were obtained from the 10 weeks old WT or parkin KO mouse liver as described previously [27]. Briefly, anesthetize the mice by injecting a mixture of Ketamine (80 mg/kg) and Xylazine (5 mg/kg) in 200 μL of saline intraperitoneally. After perfusion with Hank’s Balanced Salt Solution (HBSS)-EGTA solution (0.5 mM EGTA in HBSS, Gibco, Grand Island, NY, USA) pH = 8), collect the liver then cut it to release hepatocytes. The resulting cells were gently pressed through a 100 μm cell strainer (BD, Franklin Lakes, New Jersey). The filtered cells were washed by Dulbecco’s modified Eagle (DMEM) medium and plated into 100 mm^2^ dishes. The cells were grown at 37 °C in 5% CO2-humidified air in DMEM medium that contained 10% fetal bovine serum (FBS), 100 U/ml penicillin, and 100 mg/ml streptomycin. DMEM, penicillin, streptomycin, and FBS were purchased from Gibco Life Technologies (Grand Island, NY, USA). To confirm the role of parkin deficiency in ethanol-treated mouse primary hepatic cells, the cells from the liver of WT or parkin KO mouse were treated with ethanol (100 mM) for 24 h. To examine whether β-catenin signaling associated with hepatic lipid accumulation, cells were pre-treated with β-catenin signaling inactivator, IWR-1 (10 μM, sigma, St. Louis, MO) for 3 h, then treated with ethanol (100 mM) for 24 h.

### Oil red O staining

Liver tissues were fixed in formalin and cut by frozen section at 10 μm. Next, sections were rinsed with propylene glycol and stained with a 0.2% Oil Red O solution in propylene glycol for 30 min at room temperature, and subsequently washed with tap water. Cells were washed twice with phosphate-buffered saline (PBS), fixed with 0.5% glutaraldehyde for 3 h at room temperature, washed again with PBS, and allowed to dry completely. Next, fixed cells were stained with a 0.2% Oil Red O solution in isopropanol diluted in distilled water (6:4) for 1 h at room temperature, and subsequently washed twice with PBS. Stained lipid droplets were observed with a light microscope (Nikon, Tokyo, Japan). Stained oil droplets were dissolved with isopropanol and quantified by spectrophotometrical analysis at 500 nm.

### RNA isolation and quantitative real-time RT-PCR

Total RNA was isolated from mouse liver using Trizol (Invitrogen, Carlsbad, CA). Samples were reverse-transcribed using ProSTAR™ (Stratagene, La Jolla, CA, USA). Gene expression analysis was performed by RT-PCR using QuantiNova SYBR green PCR kit (Qiagen, Valencia, CA, USA). Information on each primer is provided in Additional file 1: Table S1 and S2.

### Histological techniques

For histological processing, liver tissues were fixed in phosphate buffer containing 10% formaldehyde and decalcified with EDTA. Fixed tissues were processed by routine methods to paraffin blocks. Specimens were sectioned at 4 μm and stained with H&E.

### Western blot analysis

Homogenized liver tissues and human hepatic cells were lysed by protein extraction solution (PRO-PREP, iNtRONBiotechnology, Korea) containing protease inhibitor cocktail (Calbiochem, Germany) and phosphatase inhibitor cocktail (Roche, Germany). Total proteins (30 μg) were separated by SDS-PAGE and transferred to a PVDF membrane (Millipore, Billerica, MA). The membrane was blocked with 5% skim milk overnight and then incubated with primary antibodies (diluted 1:1000) for 1 h at room temperature. The membranes were immunoblotted with following primary antibodies: parkin and HA (Santa Cruz Biotechnology, Dallas, Texas, USA), and β-catenin (abcam, MA, USA). After washing with Tris-buffered saline containing 0.05% Tween-20 (TBST), the membrane was incubated with horseradish peroxidase-conjugated secondary antibodies (diluted 1:3000) for 1 h at room temperature. Binding of antibodies to the PVDF membrane was detected with enhanced chemiluminescence solution (Amersham Bioscience, Buckinghamshire, UK) and X-ray film (AGFA, Belgium).

### Immunocytochemistry

Cells were fixed by acetone for 15–20 min and washed in PBS and incubated with the following antibodies: parkin and β-catenin (1:200; abcam, MA, USA); donkey anti-mouse IgG or goat anti-rabbit IgG (1:200; Jackson Laboratories). Images were acquired with a confocal microscope Zeiss LSM 710 (Zeiss, Germany).

### Immunoprecipitation

HepG2 cells were co-transfected with myc-tagged parkin, flag-tagged β-catenin and HA-tagged ubiquitin expression vector. Overnight incubation after transfection, cells were treated with 100 mM ethanol for 24 h, and then lysed in 50 mM HEPES, pH 7.5, 150 mM NaCl, 5% glycerol, 20 mM b-glycerophosphate, 0.5% NP-40, 0.1% TX-100, and 1 mM EDTA. For immunoprecipitation, cell lysates were mixed with 1 μg of HA antibody and then pulled down using 35 μl of protein G agarose beads (KPL, Gaithersburg, MD). Western blotting was performed using a myc tag antibody (Millipore-Upstate, Bedford, MA) and flag antibody (Sigma, St. Louis, MO).

### Statistical analysis

The Cell experiments were conducted in triplicate, and all experiments were repeated at least three times with similar results. The data were analyzed using the GraphPad Prism 4 version 4.03 software (Graph-Pad Software, La Jolla, CA). Data are presented as mean ± SD. The differences in all data were assessed by one-way analysis of variance. When the *P* value in the analysis of variance test indicated statistical significance, the differences were assessed by the Tukey’s test. A value of *P < 0.05, ^*#*^*P* < 0.05 was considered to be statistically significant. .

## Results

### Parkin-deficient mice exhibit attenuation of chronic ethanol-induced hepatosteatosis

**Fig. 1:**
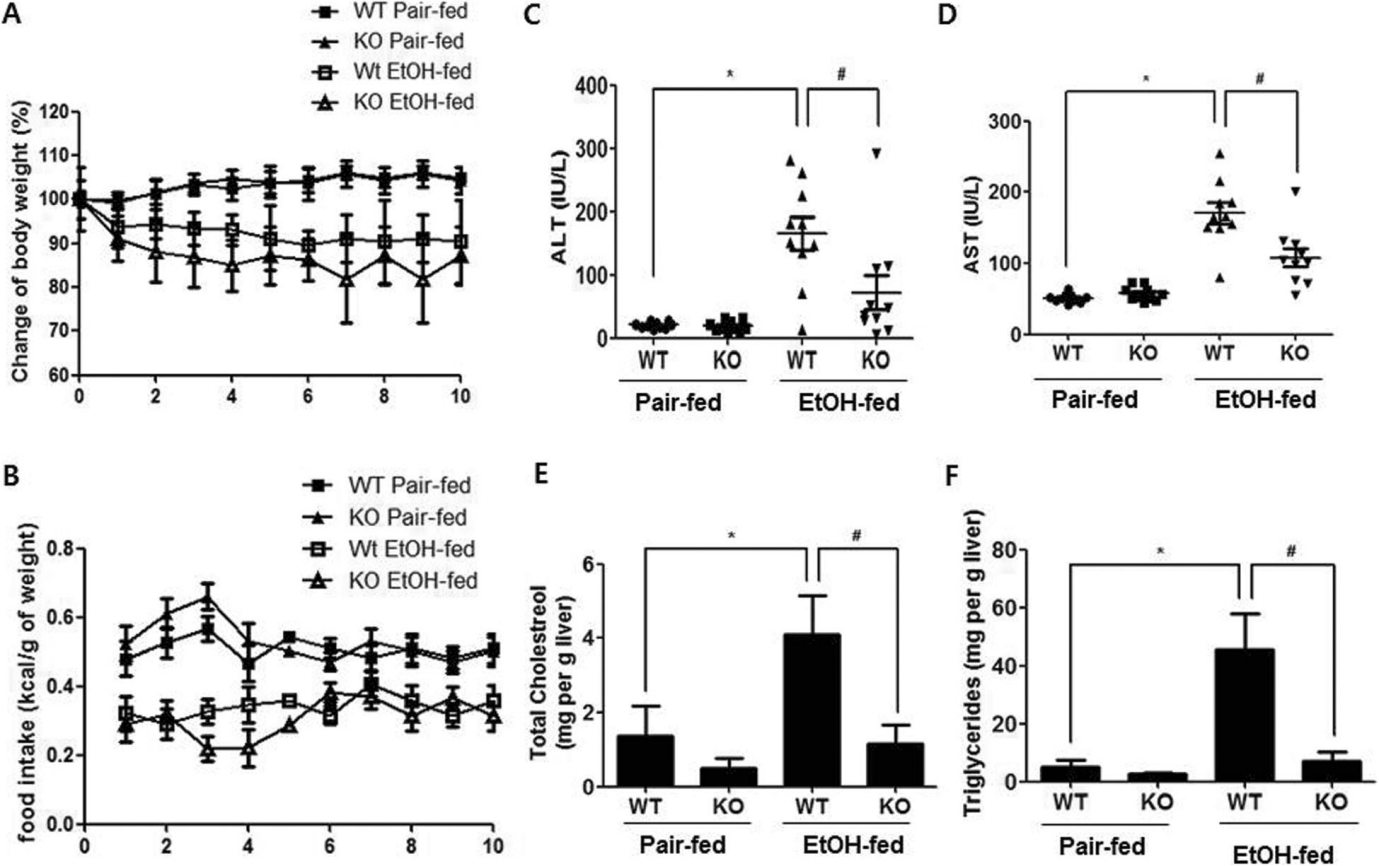
Alcoholic liver injury model induced by chronic binge-ethanol (EtOH) feeding in parkin KO mice. **a** The body weight and **b** daily food intake of WT and parkin KO mice, which were fed control or ethanol-supplemented diet for 10 days. Serum (**c**) ALT and **d** AST from WT and Parkin KO mice after 8 days of control or ethanol diet. The levels of **e** cholesterol and **f** triglycerides in the livers of control or ethanol-fed mice. *n* = 10 per group; means ± SD; ^*^P < 0.05, pair-fed WT mice versus ethanol-fed WT mice; ^#^*P* < 0.05, ethanol-fed WT mice versus ethanol-fed parkin KO mice

**Fig. 2:**
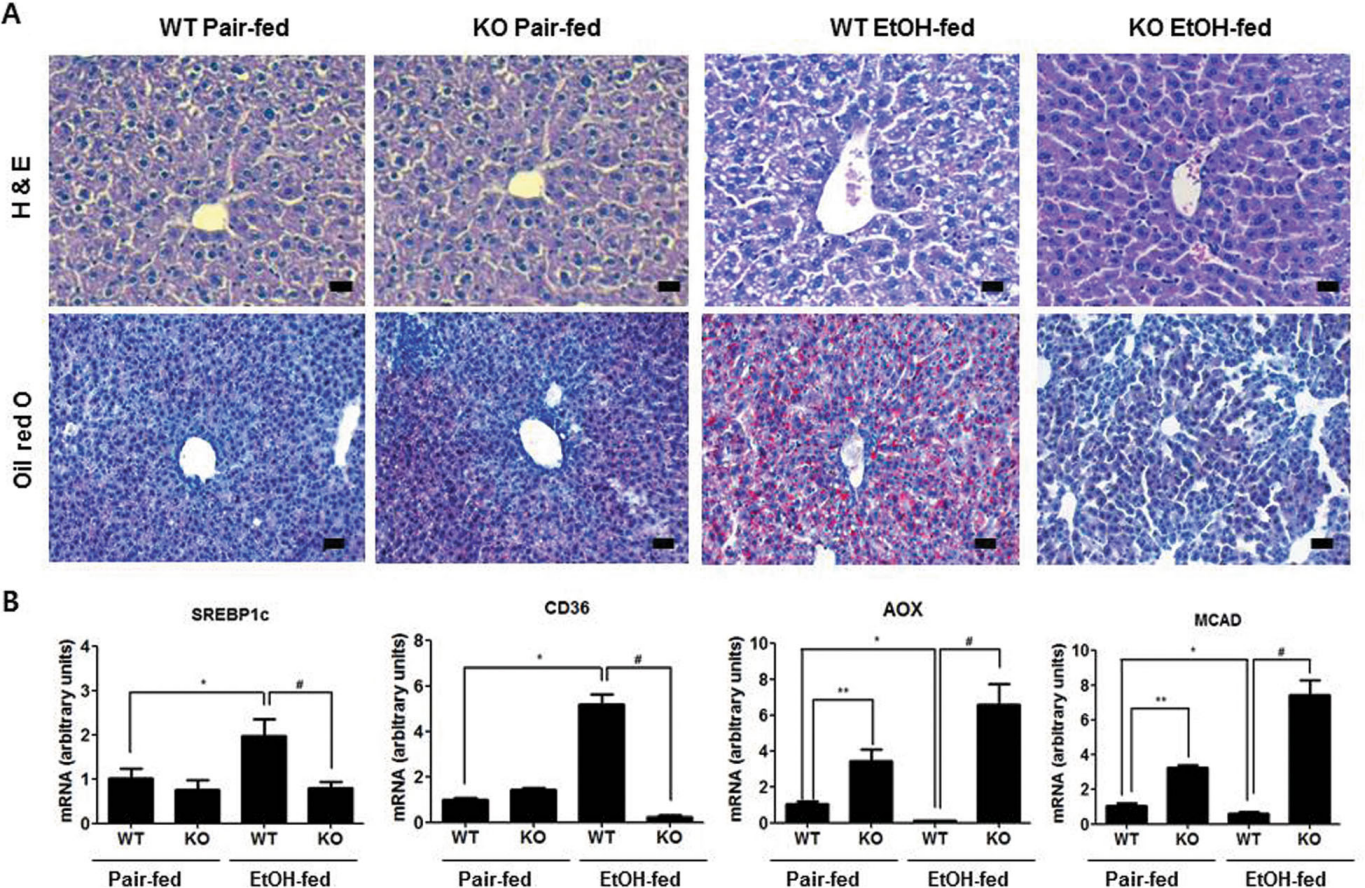
Parkin KO mice show protection against ethanol-induced hepatic lipid accumulation. **a** Liver sections of pair- or ethanol-fed WT mice and parkin KO mice were stained with hematoxylin and eosin (H&E) and Oil red O (Scale bars, 50 μm). **b** Expression of genes involved in lipogenesis (SREBP-1c and CD36) and lipid oxidation (AOX and MCAD) in the livers of pair- or ethanol-fed WT mice and parkin KO mice. *n* = 10 per group; means ± SD; ^*^P < 0.05, pair-fed WT mice versus ethanol-fed WT mice; ^#^*P* < 0.05, ethanol-fed WT mice versus ethanol-fed parkin KO mice

We examined the effects of parkin deletion on chronic ethanol-induced changes in body weight, liver cholesterol, liver triglycerides, and serum alanine aminotransferase (ALT) and aspartate aminotransferase (AST). The changes in body weight and food intake were similar between the pair-fed mice of two groups, or between the ethanol-fed mice of two groups (Fig. 1a and b). The serum ALT and AST levels in ethanol-fed parkin KO mice were lower than that in ethanol-fed wild-type (WT) mice (Fig. 1c and d). Biochemical assays also revealed higher liver triglyceride and cholesterol levels in the ethanol-fed WT group, compared with ethanol-fed parkin KO mice (Fig. 1e and f). Histopathological studies revealed vesicular hepatosteatosis in ethanol-fed WT mice, but its manifestation was attenuated in ethanol-fed parkin KO mice (Fig. 2a). Similarly, Oil red O staining for neutral lipids confirmed the increased hepatosteatosis in the livers of ethanol-fed WT mice compared to ethanol-fed parkin KO mice (Fig. 2a). In order to understand the mechanism underlying the reduced hepatic lipid accumulation in parkin KO mice, we analyzed the genetic expression of the principal factors related to lipogenesis, fatty acid uptake, and β-oxidation in the liver. Levels of mRNA expression of genes involved in lipogenesis (sterol regulatory element binding protein 1c (SREBP-1c)) and lipid uptake (CD36) were higher in the livers of ethanol-fed WT mice compared to pair-fed WT mice, but those were not increased in the livers of ethanol-fed parkin KO mice (Fig. 2b). Moreover, the mRNA expression levels of the β-oxidation-related genes acyl CoA oxidase (AOX) and medium-chain acyl-CoA dehydrogenase (MCAD) were decreased in the livers of ethanol-fed WT mice compared to pair-fed WT mice; however, their expression was increased in the livers of parkin KO mice. Since activated SREBP1 is translocated into the nucleus, we investigated quantity of SREBP1 in the nuclear fraction of the liver. SREBP1 was increased in the nuclear fraction of WT mice liver by ethanol feeding, while increased SREBP1 was lowered in in the nuclear fraction of ethanol-fed parkin KO mice liver compared to ethanol-fed WT mice (Additional file 2: Figure S1). Thus, these results suggest that parkin deficiency attenuates ethanol-induced hepatosteatosis.

### Parkin deficiency regulates the expression of genes involved in lipid metabolism

**Fig. 3:**
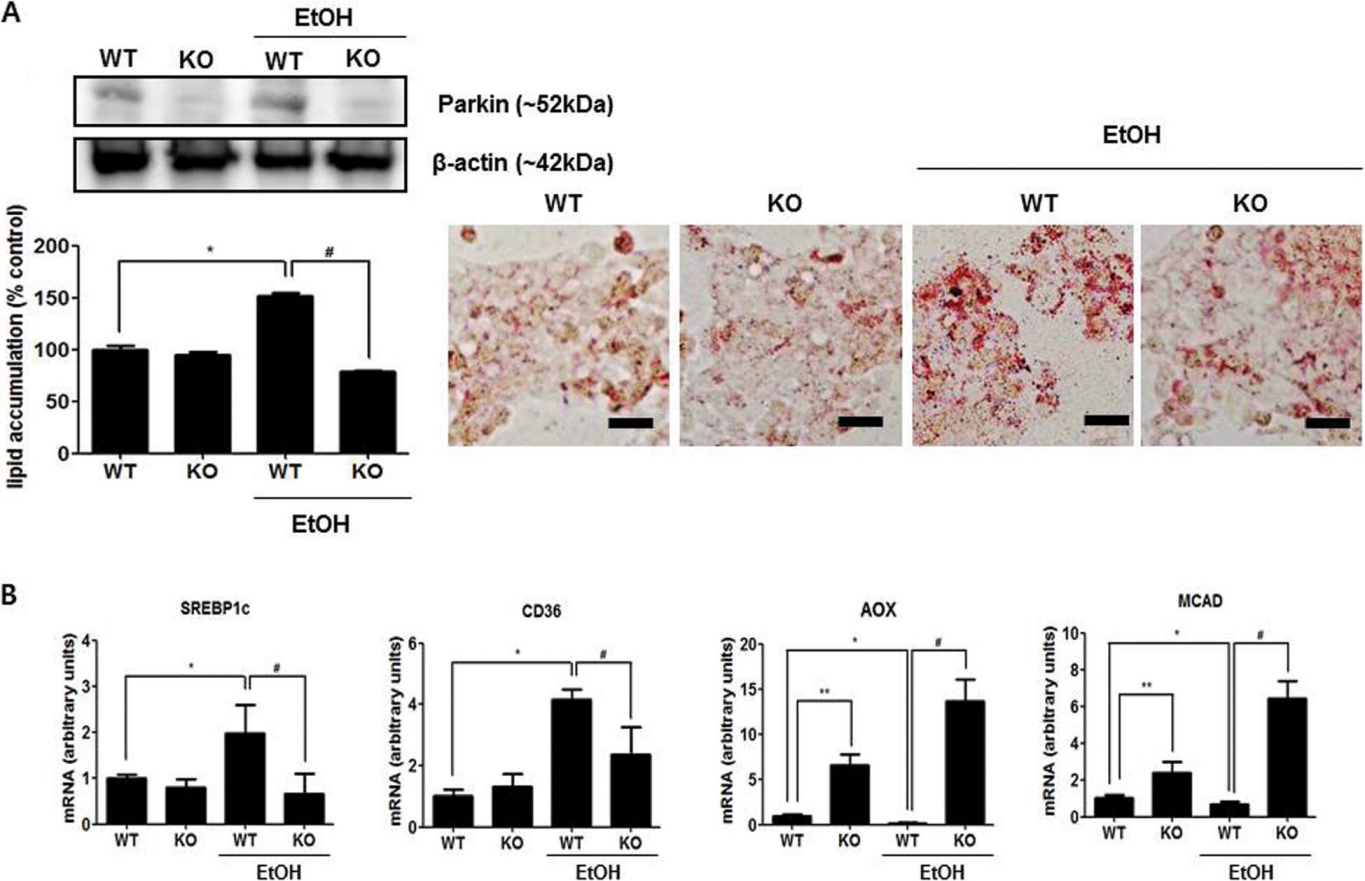
The loss of parkin decreases ethanol-induced lipid accumulation and regulates expression of genes involved in lipid metabolism in mouse hepatic cells. **a** Immunoblots of parkin and Oil red O staining for lipid accumulation in primary hepatic cells from WT or parkin KO mice treated with ethanol (100 mM) or without. **b** Expression of genes involved in lipid metabolism in primary hepatic cells from WT or parkin KO mice treated with ethanol (100 mM) or without ethanol (Scale bars, 50 μm). Values are expressed as the mean ± SD of three different experiments conducted in triplicate. ^*^P < 0.05, WT versus WT treated with ethanol; ^**^P < 0.05, WT versus KO; ^#^*P* < 0.05, WT treated with ethanol versus KO treated with ethanol

Because parkin-deficient mice showed decreased lipid accumulation induced by ethanol diet in the liver, we investigated the effect of parkin deficiency in ethanol-treated mouse primary hepatic cells. We collected hepatocytes from WT or parkin KO mice and confirmed the expression of parkin by western blotting (Fig. 3a). To examine the effect of parkin deficiency on lipid accumulation in mouse primary hepatic cells induced by ethanol, we evaluated the amount of lipid accumulation through Oil red O staining in mouse hepatic cells treated with 100 mM ethanol. These experiments revealed that hepatic cells from WT mice exhibited increased lipid accumulation after ethanol treatment, whereas hepatic cells from parkin KO mice showed no change in lipid accumulation after ethanol treatment (Fig. 3a). The mRNA expression of the principal factors related to fatty acid uptake or synthesis, SREBP-1c and CD36, were downregulated in ethanol-treated hepatic cells from parkin KO mice, as compared to those in ethanol-treated hepatic cells from WT mice (Fig. 3b). The mRNA expression levels of the β-oxidation-related genes AOX and MCAD were increased in hepatic cells from parkin KO mice compared to that in hepatic cells from WT mice (Fig. 3b). The mRNA expression levels of AOX and MCAD were decreased by ethanol in hepatic cells from WT mice; however, their expression was further increased by ethanol in hepatic cells from parkin KO mice, unlike hepatic cells from WT mice (Fig. 3b).

### Parkin regulates β-catenin protein levels through interaction with β-catenin

**Fig. 4:**
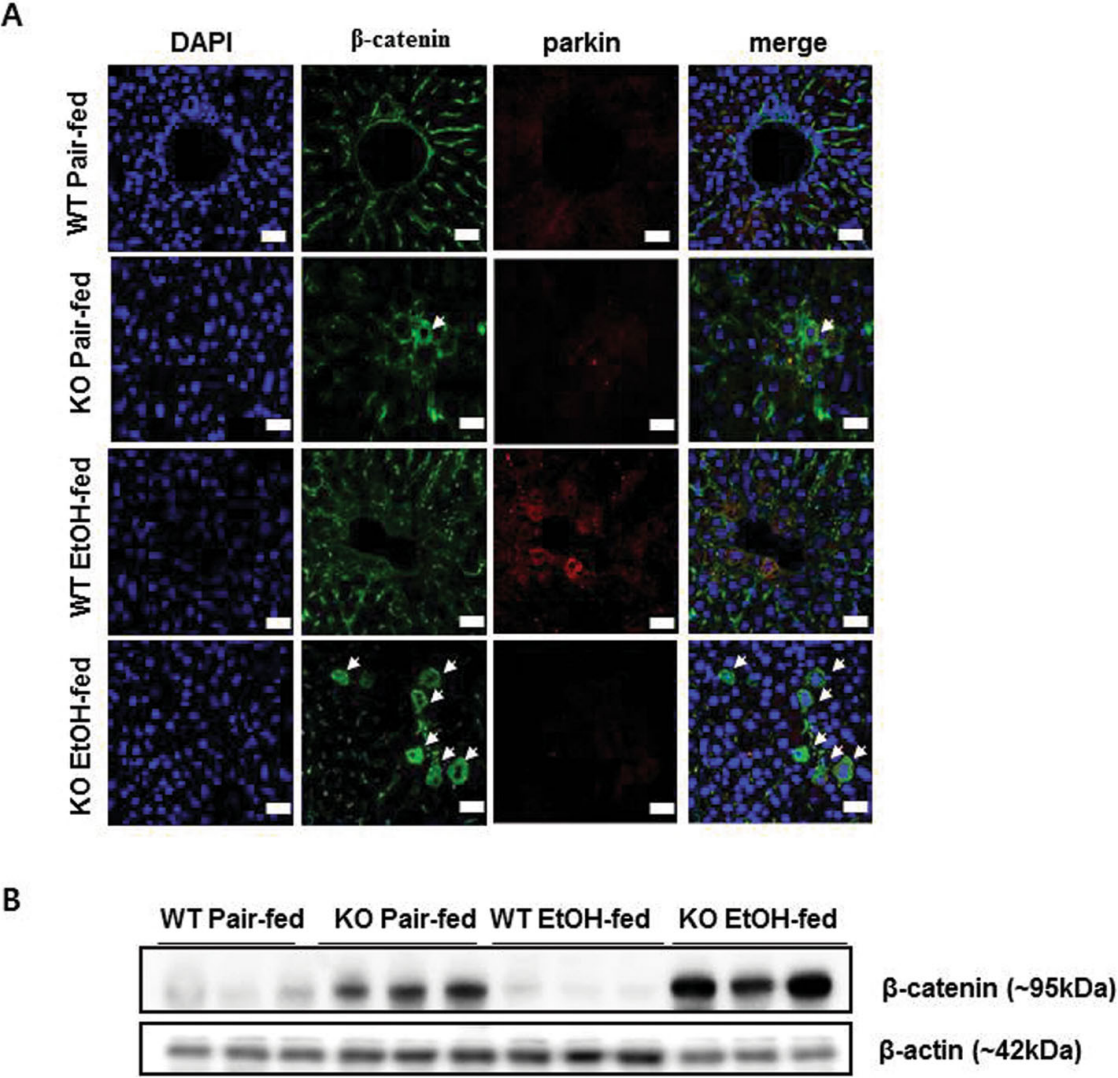
Ethanol-fed parkin KO mice show β-catenin accumulation in the liver. **a** Liver sections were triple-stained with β-catenin antibody (Alexa 488; green, arrowahead), parkin antibody (Alexa 568; red), and DAPI (nuclear counterstain; blue), and images were analyzed by confocal laser-scanning microscopy (Scale bars, 50 μm). **b** Immunoblots of β-catenin in the livers of pair- or ethanol-fed WT mice and parkin KO mice

**Fig. 5:**
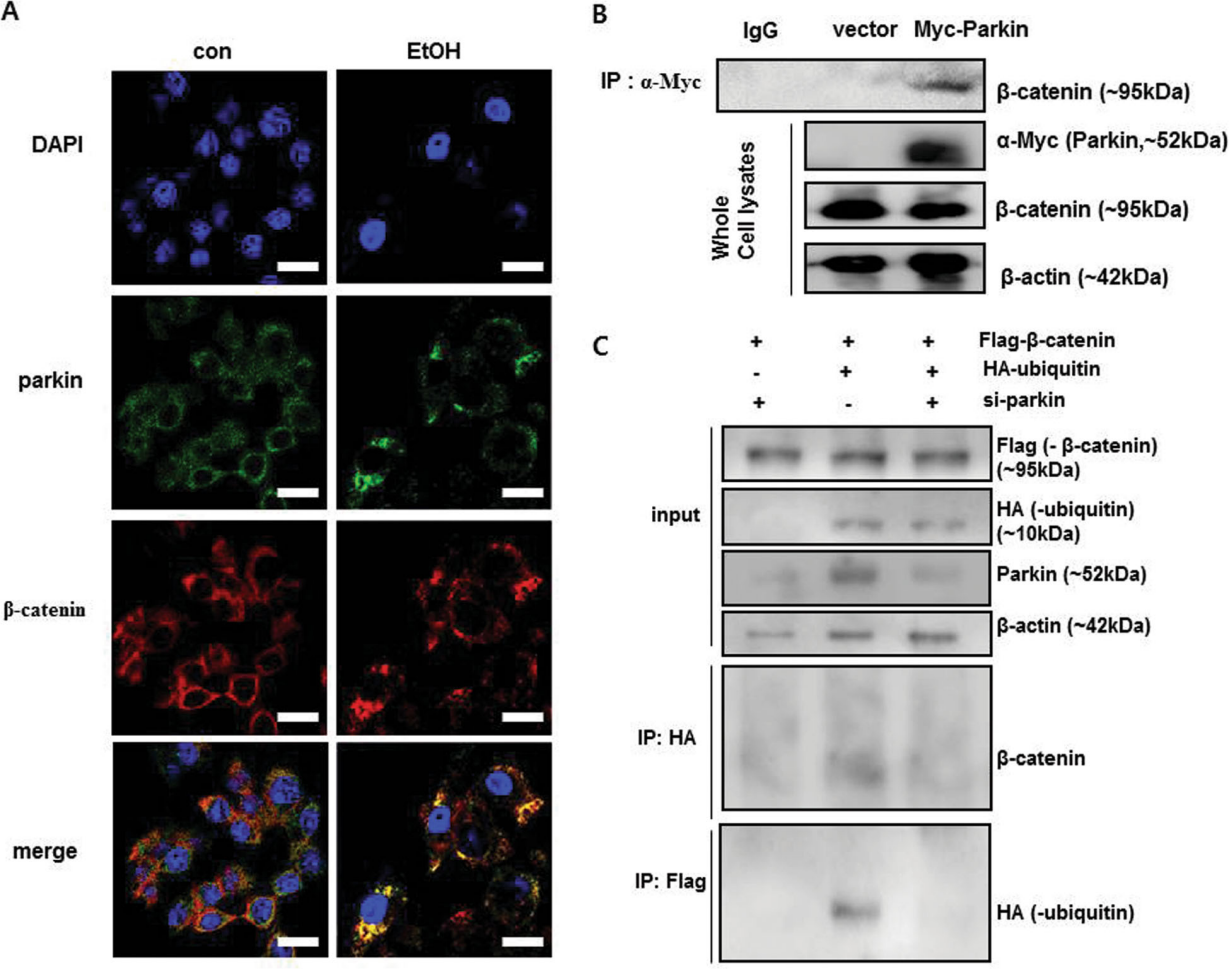
Parkin regulates β-catenin by ubiquitination in ethanol-treated hepatic cells. **a** Confocal microscopy assay of primary hepatic cells from WT mice treated with ethanol or without ethanol. Cells were triple-stained with β-catenin antibody (Alexa 488; green), parkin antibody (Alexa 568; red), and DAPI (nuclear counterstain; blue) (Scale bars, 50 μm). **b** Interaction between parkin and β-catenin. HepG2 cells were transfected with Myc-vector or Myc-parkin. After ethanol treatment for 24 h, protein extracts were immunoprecipitated with anti-Myc agarose. **c** Ubiquitination of β-catenin following immunoprecipitation (IP) with an anti-HA antibody and immunoblot analysis for β-catenin, and IP with an anti-Flag antibody and immunoblot analysis for HA-ubiquitin. Flag-β-catenin-overexpressing HepG2 cells were transfected with HA-ubiquitin and/or parkin siRNA

Because parkin interacts with β-catenin in dopaminergic neurons [23] and β-catenin protects against alcohol-mediated hepatosteatosis as well as regulates energy balance in mouse liver [6, 12], we investigated the relationship between parkin and β-catenin in liver tissue and hepatic cells. In mouse liver, we observed that β-catenin was accumulated in hepatocytes of ethanol-fed parkin KO mice, but not in that of ethanol-fed WT mice (Fig. 4a). Because parkin is a component of the E3 ubiquitin ligase complex and is involved in degradation of target proteins through ubiquitination, we investigated the protein level of β-catenin in the livers of pair-fed and ethanol-fed groups. We observed that the β-catenin protein level was increased in the livers of pair-fed parkin KO mice compared to pair-fed WT mice (Fig. 4b). The β-catenin protein level was decreased in the livers of ethanol-fed WT mice compared to that of pair-fed WT mice; however, it was increased in the livers of ethanol-fed parkin KO mice compared to that of pair-fed parkin KO mice (Fig. 4b). Next, we investigated β-catenin signaling in liver tissue. Levels of mRNA expression of β-catenin directly target genes such as cyclin D1(CCND1), peroxisome proliferator activated receptor delta (PPARD) and Axin2 were lower in the livers of ethanol-fed WT mice compared to pair-fed WT mice, but those were robustly increased in the livers of ethanol-fed parkin KO mice compared to ethanol-fed WT mice (Additional file 3: Figure S2). These results suggest that β-catenin signaling was abrogated by ethanol feeding in the liver of WT mice but it was enhanced in the liver of ethanol-fed parkin KO mice by β-catenin accumulation. To further examine whether parkin modulates β-catenin in hepatic cells, we performed confocal microscopy analysis in primary mouse hepatic cells treated with ethanol or without. We found that the parkin protein was predominantly in the cytoplasm and overlapped with endogenous cytoplasmic β-catenin in mouse hepatic cells after ethanol treatment (Fig. 5a). We then investigated whether parkin functionally interacts with β-catenin. Because DNA transfecting into primary mouse hepatic cells is indeed very difficult, thus we used HepG2 cells which are human hepatic cancer cell line in Co-immunoprecipitation (Co-IP) experiments. Co-IP of Myc-tagged parkin and subsequent immunoblot analysis for β-catenin supported the physical interaction between parkin and β-catenin (Fig. 5b). To evaluate whether parkin knockdown affects β-catenin ubiquitination, we simultaneously transfected HepG2 cells with Flag-tagged β-catenin, HA-tagged ubiquitin, and parkin siRNA and used the antibody directed against Flag-tag or HA-tag in the Co-IP assay. We observed by immunoblot analysis that β-catenin was not ubiquitinated when parkin siRNA was transfected and ubiquitin was overexpressed (Fig. 5c).

### The inhibition of β-catenin signaling ehances ethanol-mediated lipid accumulation in parkin deficient hepatocytes

**Fig. 6:**
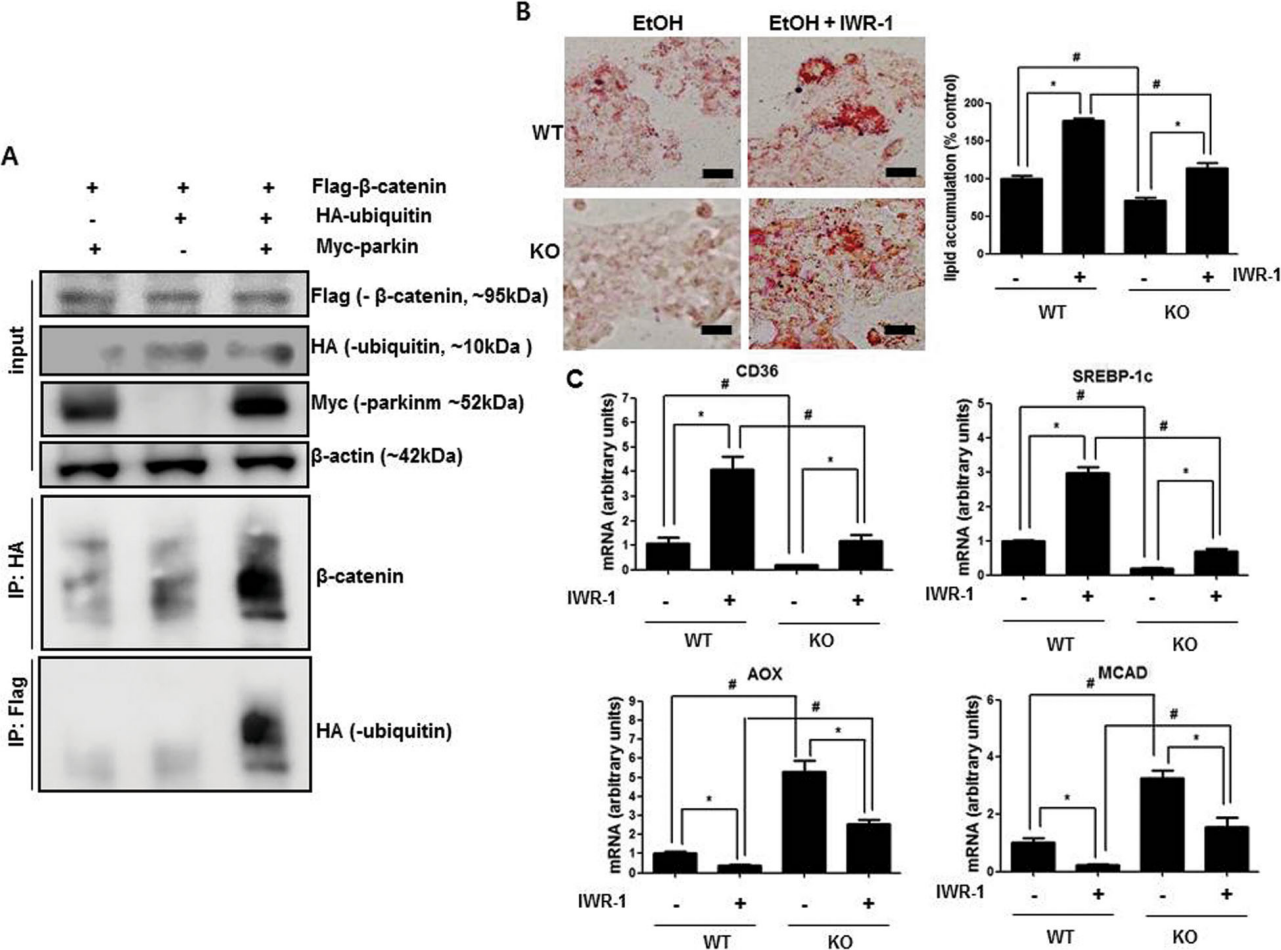
Effects of β-catenin signaling inhibitor on lipid accumulation in hepatic cells. **a** Ubiquitination of β-catenin following immunoprecipitation (IP) with an anti-HA antibody and immunoblot analysis for β-catenin, and IP of an anti-Flag antibody and immunoblot analysis for HA-ubiquitin. Flag-β-catenin-overexpressing HepG2 cells were transfected with HA-ubiquitin and/or Myc-parkin. **b** Hepatic lipid accumulation in primary hepatic cells from WT or parkin KO mice pretreated with IWR-1 (40 μM) and then treated with ethanol (100 mM) (Scale bars, 50 μm). **c** Expression of genes involved in lipid metabolism in primary hepatic cells from WT or parkin KO mice pretreated with IWR-1 (40 μM) and then treated with ethanol (100 mM). Values are expressed as the mean ± SD of three different experiments conducted in triplicate. ^*^P < 0.05, pretreated with IWR-1 versus without; ^#^*P* < 0.05, WT versus KO

Because β-catenin is a critical factor in alcohol-mediated hepatosteatosis [10–12], and we found that parkin knockdown abrogates β-catenin ubiquitination in HepG2 cells, we investigated whether parkin directly ubiquitinates β-catenin and a β-catenin signaling inhibitor abrogates the effects of parkin deficiency in ethanol-treated hepatic cells. Firstly, to evaluate whether parkin directly ubiquitinates β-catenin, we concurrently transfected HepG2 cells with Flag-tagged β-catenin, Myc-tagged parkin, and HA-tagged ubiquitin and used the antibody directed against Flag-tag or HA-tag in the Co-IP assay. Immunoblot analysis shows that parkin increased the ubiquitination of β-catenin when parkin and ubiquitin were concurrently overexpressed (Fig. 6a). Next, we evaluated the amount of lipid accumulation in hepatic cells from WT or parkin KO mice pretreated with the β-catenin signaling inhibitor IWR-1 (10 μM), and then treated with ethanol (100 mM). In hepatic cells from WT mice, ethanol-induced lipid accumulation was further increased by IWR-1 (Fig. 6b). However, ethanol-induced lipid accumulation was decreased in hepatic cells from parkin KO mice, and those decreased levels were increased by IWR-1 (Fig. 6b). In addition, we observed that the ethanol-induced mRNA expression levels of SREBP1 and CD36 were robustly increased by IWR-1 (Fig. 6c) while those mRNA expression levels were slightly increased by IWR-1 in the hepatic cells from parkin KO mice (Fig. 6c). In case of the mRNA expression levels of AOX and MCAD were decreased by IWR-1 in ethanol-treated hepatic cells from WT mice and those mRNA expression levels were higher in ethanol-treated hepatic cells from parkin KO mice compared to ethanol -treated hepatic cells from WT mice (Fig. 6c). In addition, we evaluated the amount of lipid accumulation in parkin knock down human hepatic Huh7 cells transfected with β-catenin si-RNA, and then treated with ethanol (100 mM). Like IWR-1 results, ethanol-induced lipid accumulation was further increased by β-catenin si-RNA transfection, and ethanol-induced lipid accumulation was decreased in parkin siRNA transfected Huh7 cells compared to control, and those decreased levels were increased by β-catenin si-RNA co-transfection (Additional file 4: Figure S3A). Moreover, the ethanol-induced mRNA expression levels of SREBP1 and CD36 were also robustly increased by β-catenin si-RNA transfection while those mRNA expression levels were slightly increased by β-catenin si-RNA transfection in the parkin si-RNA transfected Huh7 cells (Additional file 4: Figure S3B). The mRNA expression levels of AOX and MCAD were decreased by β-catenin si-RNA transfection in ethanol-treated control Huh7 cells as well as ethanol-treated Huh7 cells transfected with parkin si-RNA and those mRNA expression levels were slightly higher in ethanol-treated Huh7 cells co-transfected with parkin si-RNA and β-catenin si-RNA compared to ethanol-treated Huh7 cells transfected with β-catenin si-RNA (Additional file 4: Figure S3B).

### The β-catenin signaling inhibitor IWR-1 abrogates the attenuation effect of ethanol-mediated hepatosteatosis in parkin KO mouse liver

**Fig. 7:**
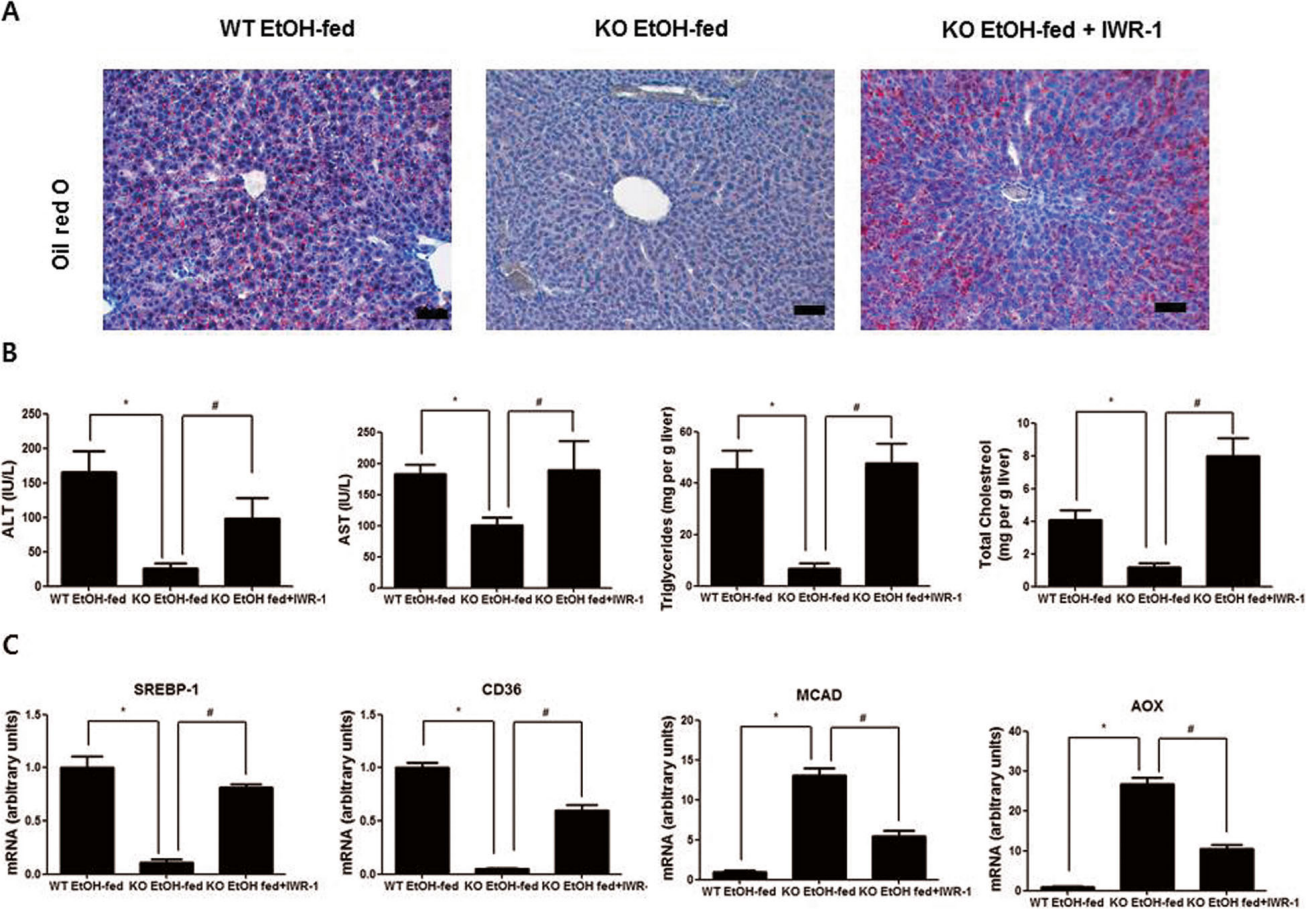
Effects of β-catenin signaling inhibitor on hepatic lipid accumulation in ethanol-fed WT and parkin KO mice. **a** Liver sections of ethanol-fed WT mice, ethanol-fed parkin KO mice, and ethanol-fed Parkin KO mice injected with IWR-1 (25 μmol/kg) were stained with Oil red O (Scale bars, 50 μm). **b** Serum ALT and AST and liver cholesterol and triglycerides from ethanol-fed WT mice, ethanol-fed parkin KO mice, and ethanol-fed parkin KO mice injected with IWR-1 (25 μmol/kg). **c** Expression of genes involved in lipogenesis (SREBP-1c and CD36) and lipid oxidation (AOX and MCAD) in the livers of ethanol-fed WT mice, ethanol-fed parkin KO mice, and ethanol-fed parkin KO mice injected with IWR-1 (25 μmol/kg). *n* = 10 per group; means ± SD; ^*^P < 0.05, ethanol-fed WT mice versus ethanol-fed parkin KO mice; ^#^*P* < 0.05, ethanol-fed parkin KO mice versus ethanol-fed parkin KO mice injected with IWR-1

Because the β-catenin signaling inhibitor IWR-1 blocked the effect of parkin deficiency in ethanol-treated HepG2 cells, we investigated the effect of IWR-1 on lipid accumulation in the livers of ethanol-fed parkin KO mice with i.p. injection of IWR-1 (25 μmol/kg). We observed that ethanol-induced hepatic lipid accumulation was decreased in the livers of ethanol-fed parkin KO mice compared to ethanol-fed WT mice, and this decreased hepatic lipid accumulation was reversed by IWR-1 administration in the livers of ethanol-fed parkin KO mice (Fig. 7a). Moreover, the serum ALT and AST levels as well as liver triglyceride and total cholesterol levels were decreased in ethanol-fed parkin KO mice compared to those in ethanol-fed WT mice; however, those levels were increased by IWR-1 administration in ethanol-fed parkin KO mice (Fig. 7b). We also observed that mRNA expression levels of SREBP-1c and CD36 were downregulated in the livers of ethanol-fed parkin KO mice compared to that in the livers of ethanol-fed WT mice, and those levels were restored by IWR-1 administration in ethanol-fed parkin KO mice (Fig. 7c). The mRNA expression levels of AOX and MCAD were increased in the livers of ethanol-fed parkin KO mice compared to that in the livers of ethanol-fed WT mice, and those levels were restored by IWR-1 administration in ethanol-fed parkin KO mice (Fig. 7c).

## Discussion

As *PARK2* mutation is associated with juvenile PD, many studies have published its function in neuronal disease and development. Cigarette smoking and coffee consumption are associated with lower risk of PD [28], but the relationship between alcohol abuse and PD remains unclear. In several studies, alcohol consumption was not associated with PD [29, 30], but other research on alcohol consumption and PD risk showed conflicting data. Liu et al. reported that total alcohol consumption is not related to PD risk but moderate beer drinkers showed lower PD risk and heavy liquor drinkers displayed higher PD risk; thus, the type of alcohol consumption may have different effects on PD risk [31]. Although the relationship between PD and alcoholic consumption remains unclear, recently data suggest that *PARK2* mutation is associated with metabolic disease because parkin expression shows the highest level in metabolically active tissues [32]. Williams et al. reported that parkin-deficient mice showed increased hepatic steatosis compared to WT mice after acute ethanol consumption [19]. However, other studies reported opposing results. Pesah et al. reported that *Drosophila* with genetically disrupted parkin showed diminished body mass [33]. Kim et al. reported that parkin deficient mice exhibited attenuation of high fat diet-induced hepatic lipid accumulation [22]. Consistent with these studies, we also showed that chronic ethanol-induced fatty liver was attenuated in parkin deficient mice. Thus, we investigated the mechanism of parkin in lipid accumulation by chronic ethanol consumption.

Body weight change and food intake were not significantly different between ethanol-fed WT mice and ethanol-fed parkin KO mice. However, the serum ALT and AST and liver cholesterol and triglycerides were lower, and hepatosteatosis was more attenuated in ethanol-fed parkin KO mice than in ethanol-fed WT mice. Additionally, we observed that the expression of transcripts involved in fatty acid uptake and synthesis was lower in the livers of ethanol-fed parkin KO mice compared to that in the livers of ethanol-fed WT mice. Moreover, the expression of transcripts involved in lipid β-oxidation was higher in the livers of ethanol-fed parkin KO mice compared to that in the livers of ethanol-fed WT mice. In addition, we found that primary hepatic cells from parkin KO mice also showed attenuation of ethanol-induced lipid accumulation. These results suggest that parkin deficiency attenuates chronic ethanol-induced fatty liver damage by decreasing hepatic lipid accumulation.

The E3 ubiquitin-ligase complex promotes proteasomal degradation of substrate proteins and/or modifies protein function and stability by ubiquitination. Several studies have shown that E3 ubiquitin ligase enzymes modulate lipid biology [34] and protect against metabolic disorder and insulin resistance by excessive fat consumption [35, 36]. Parkin is an E3 ubiquitin ligase expressed in several organs and multiple substrates have been identified [15]. Rawal et al. reported that parkin interacts with β-catenin and protects against cell death by excessive β-catenin signaling through β-catenin degradation in neuronal cells [23]. However, the function of parkin in hepatic lipid accumulation through interaction with β-catenin has not been studied. In several studies, β-catenin was closely associated with hepatic metabolism and hepatocellular energy balance. Behari et al. reported that liver-specific β-catenin knockout mice exhibit increased susceptibility to diet-induced hepatosteatosis [11]. Liver-specific β-catenin knockout mice also show abnormal bile canalicular morphology, low bile flow rates and intrahepatic cholestasis [37]. Furthermore, Liu et al. reported that β-catenin knockout mice develop severe hepatosteatosis after ethanol consumption [12]. Ethanol-induced β-catenin knockout mice exhibit abnormal mitochondrial function and down-regulation of genes involved in fatty acid β-oxidation such as MCAD and AOX in the liver [38]. These results suggest that β-catenin is an essential factor for alcohol-mediated hepatosteatosis. In our data, we found that β-catenin was accumulated and the expression of β-catenin directly genes were increased in the liver of ethanol-fed parkin KO mice compared to ethanol-fed WT mice. We also observed that primary hepatic cells from parkin KO mice exhibited attenuation of ethanol-induced hepatic lipid accumulation. Similar to parkin’s interaction with β-catenin in neuronal cells, we also observed that β-catenin interacts with parkin, and it was degraded by ubiquitination in HepG2 human hepatic cells; however, it did not interact with ubiquitin in parkin siRNA-transfected cells. These results suggest that parkin modulates β-catenin signaling by ubiquitination. In primary hepatic cells from parkin KO treated with ethanol, decreased hepatic lipid accumulation, down-regulated mRNA expressions of genes involved in fatty acid uptake and lipogenesis, and up-regulated mRNA expressions of genes involved in β-oxidation were detected compared to primary hepatic cells from WT mice. These results mediated by parkin deficiency were inhibited by the β-catenin signaling inhibitor IWR-1 or β-catenin si-RNA transfection. Similar to the in vitro data, IWR-1 administration also abrogated the protective effects of parkin deficiency on ethanol-induced fatty liver in parkin KO mice. These results suggest that parkin deficiency attenuates ethanol-induced hepatic lipid accumulation through β-catenin signaling by a defect in β-catenin degradation.

## Conclusion

In summary, we show here that loss of parkin inhibit hepatic lipid accumulation in the fatty liver by chronic ethanol consumption. Although parkin deficiency exacerbates liver injury by acute ethanol consumption, it leads to defective β-catenin degradation and results in inhibition of hepatic lipid accumulation under chronic ethanol consumption condition. Thus, parkin may play a role in hepatic lipid metabolism through regulation of β-catenin signaling in chronic fatty liver disease.

## Additional files

Additional file 1: **Table S1.** Mouse oligonucleotide primers used for real time RT-PCR. **Table S2.** Human oligonucleotide primers used for real time RT-PCR. (DOC 34 kb)

Additional file 2: **Figure S1.** Effects of parkin deficiency on SREBP1 activation in chronic plus binge ethanol feeding mice model. (TIF 1188 kb)

Additional file 3: **Figure S2.** Effects of parkin deficiency on β-catenin signaling in chronic plus binge ethanol feeding mice model. (TIF 1258 kb)

Additional file 4: **Figure S3.** Effects of β-catenin siRNA on lipid accumulation in ethanol-treated human hepatic Huh7 cells. (ZIP 3603 kb)

## Data Availability

Not applicable.

## Abbreviations

ALT: Alanine transaminase
AOX: Acyl CoA oxidase
AST: Aspartate transaminase
IP: Immunoprecipitation
MCAD: Medium chain acyl CoA dehydrogenase
parkin KO mice: parkin knockout mice
PD: Parkinson’s disease,
SREBP-1c: Sterol regulatory element binding protein 1c
WT mice: Wild-type mice
