# Supplementary material for: Parkin deficiency prevents chronic ethanol-induced hepatic lipid accumulation through β-catenin accumulation
Source: Cell Commun Signal. 2019 Aug 22;17:104. doi: 10.1186/s12964-019-0424-5 (PMC6704582; doi:10.1186/s12964-019-0424-5)
Supplement: Supplementary file 1 — Table S1. Mouse oligonucleotide primers used for real time RT-PCR. Table S2. Human oligonucleotide primers used for real time RT-PCR. (DOC 34 kb) [file 12964_2019_424_MOESM1_ESM.doc]

**Table S1.** Mouse oligonucleotide primers used for real time RT-PCR.

| Target gene | Sequences | |
| --- | --- | --- |
|  | Forward (5’-3’) | Reverse (5’-3’) |
| ACTB  SREBP1  CD36  AOX  MCAD  CCND1  PPARD  Axin2 | GGCTGTATTCCCCTCCATCG  CAAGGCCATCGACTACATCCG  GGAGCCATCTTTGAGCCTTCA  GAGCAGCAGGAGCGTTTCTT  CCAGAGAGGAGATTATCCCCG  GCGTACCCTGACACCAATCTC  GCAGCCTCAACATGGAATGTC  AACCTATGCCCGTTTCCTCTA | CCAGTTGGTAACAATGCCATGT  CACCACTTCGGGTTTCATGC  GAACCAAACTGAGGAATGGATCT  CAGGACTATCGCATGATTGGAAG  TACACCCATACGCCAACTCTT  CTCCTCTTCGCACTTCTGCTC  GAGCTTCATGCGGATTGTCC  GAGTGTAAAGACTTGGTCCACC |

**Table S2.** Human oligonucleotide primers used for real time RT-PCR.

| Target gene | Sequences | |
| --- | --- | --- |
|  | Forward (5’-3’) | Reverse (5’-3’) |
| ACTB  SREBP1  CD36  AOX  MCAD | CATGTACGTTGCTATCCAGGC  CGGAACCATCTTGGCAACAGT  CTTTGGCTTAATGAGACTGGGAC  GGAACTCACCTTCGAGGCTTG  GGAAGCAGATACCCCAGGAAT | CTCCTTAATGTCACGCACGAT  CGCTTCTCAATGGCGTTGT  GCAACAAACATCACCACACCA  TTCCCCTTAGTGATGAGCTGG  AGCTCCGTCACCAATTAAAACAT |
