# Supplementary figures and images for: Parkin deficiency prevents chronic ethanol-induced hepatic lipid accumulation through β-catenin accumulation
Source: Cell Commun Signal. 2019 Aug 22;17:104. doi: 10.1186/s12964-019-0424-5 (PMC6704582; doi:10.1186/s12964-019-0424-5)

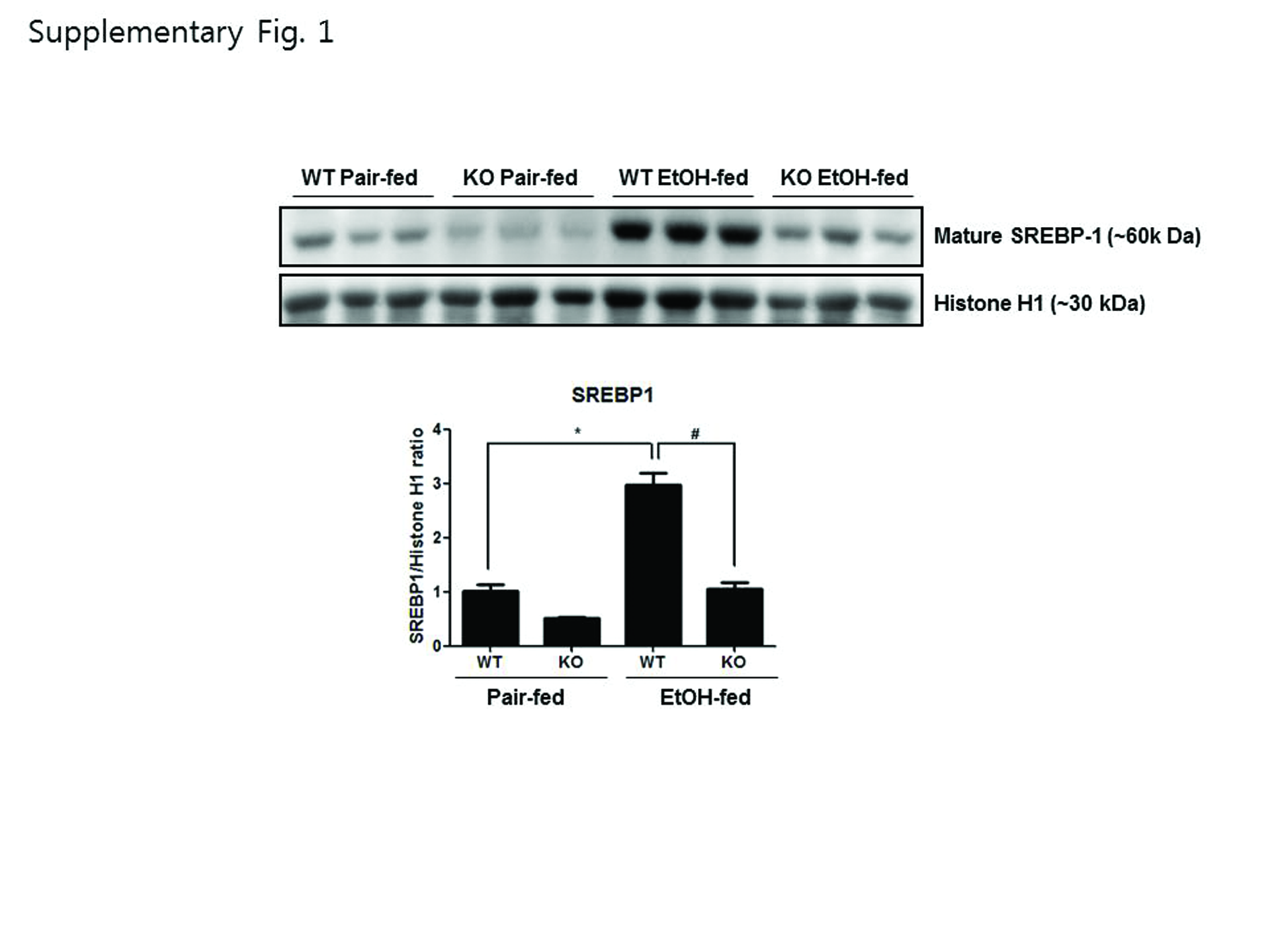

Supplement: Supplementary file 2 — Figure S1. Effects of parkin deficiency on SREBP1 activation in chronic plus binge ethanol feeding mice model. (TIF 1188 kb) [file 12964_2019_424_MOESM2_ESM.tif]

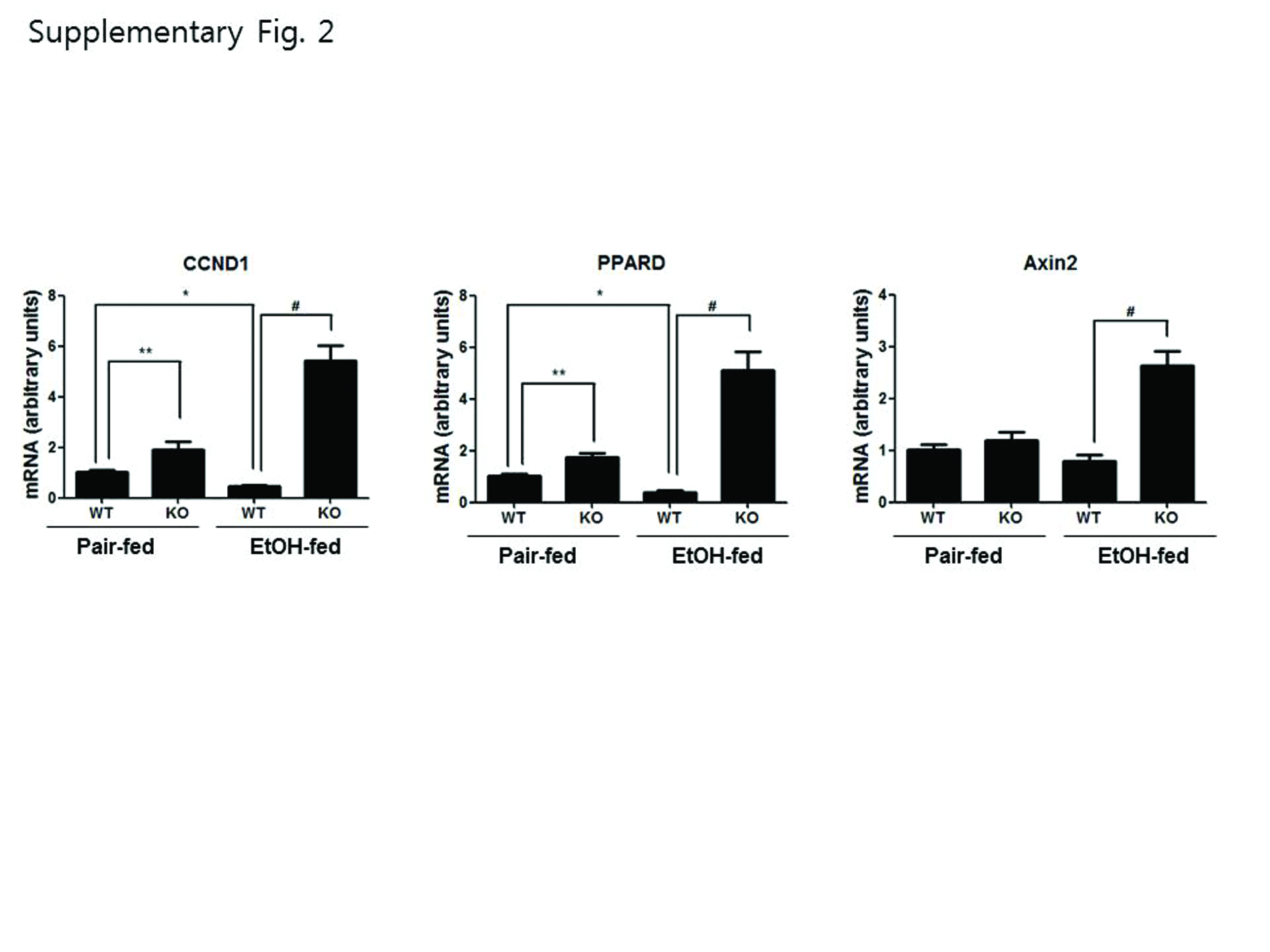

Supplement: Supplementary file 3 — Figure S2. Effects of parkin deficiency on β-catenin signaling in chronic plus binge ethanol feeding mice model. (TIF 1258 kb) [file 12964_2019_424_MOESM3_ESM.tif]

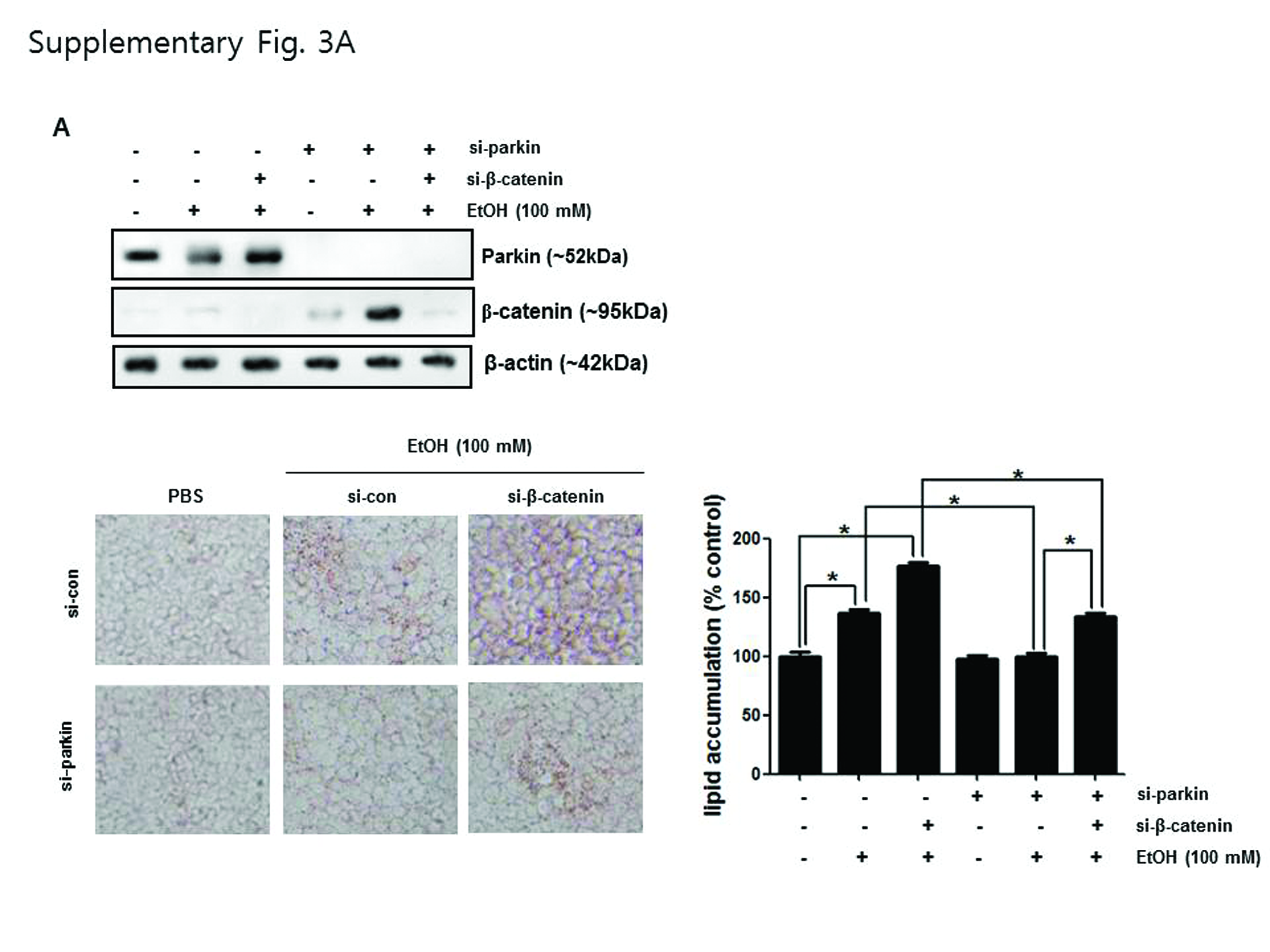

Supplement: Supplementary file 4 — Figure S3. Effects of β-catenin siRNA on lipid accumulation in ethanol-treated human hepatic Huh7 cells. (ZIP 3603 kb) [file 12964_2019_424_MOESM4_ESM.zip › Supplementary Fig 3A.tif]

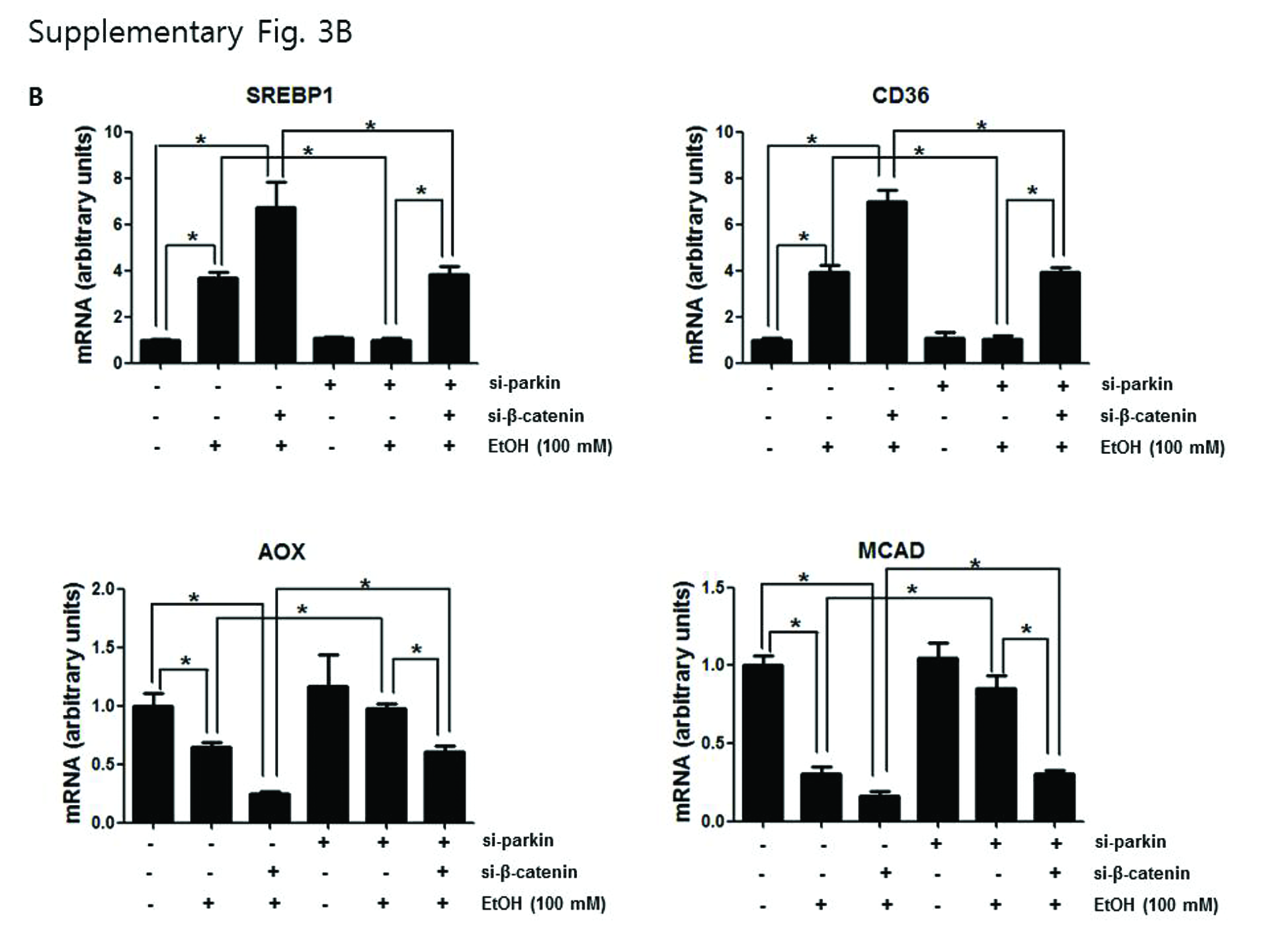

Supplement: Supplementary file 4 — Figure S3. Effects of β-catenin siRNA on lipid accumulation in ethanol-treated human hepatic Huh7 cells. (ZIP 3603 kb) [file 12964_2019_424_MOESM4_ESM.zip › Supplementary Fig 3B.tif]
